# Supplementary material for: Clinical anemia predicts dermal parasitism and reservoir infectiousness during progressive visceral leishmaniosis
Source: PLoS Negl Trop Dis. 2024 Nov 8;18(11):e0012363. doi: 10.1371/journal.pntd.0012363 (PMC11578447; doi:10.1371/journal.pntd.0012363)
Supplement: S1 Fig — Leishmania infantum amastin and canine CD14 RNAscope probes. (DOCX) [file pntd.0012363.s001.docx]

***L. infantum* amastin (LINF_080011900, predicted RNA/mRNA sequence):** ATGGCGTGCAAGCTCGGCGTCATTATCTACGTCGTCCTCCAGTTCATCGCGTTCTTCTCCGTGCTGATCGGTACGGGGGTCGACATGTTTTACATCAAGCCGGAGCACAGCTTTGGCGCCAGGGTATGCATAACCCTGTGGGGTGGAAAGACTGACTGTCGAAAAGCCAAGGTAACCATCACCCCGGGCGTACGGTGGAAGTTCTGCCCCATCCGCCTCAGAAACTTCCGCATTGGTGAGGCGTTCGCTGTCATCTCCATCTTCGTGTACGGCGCGGCGTTCCTCTTCGGCTTCCTTTTGCTGTACTGCTGCGCTGGCTTCCGCTGGCTCTGCCTGGCGCTGAACATCGTGGGCGCTGTCACCGCTTGCGTTGTCTGGGCGGTCATGGTGGTCACCTACAGACTCCCAGAGCCCAAGTGCCTGGAGCTGAGTGACGGCTACGATTTCGGCACCGGCTTCGGTCTCTTCGTGCTTGCCTGGATCCTGGATATCATCGACATTATCTTCCTGATGCTCCCGTGGCAAATCGGAGAGTTCGGTGAGGGTGACGAACCGAATGGGCAGGAGGAGGAGGAGGAGGAGGTGGTGCAGTCTAAAAAAGCAACGGAGGAGTAG

**Canine CD14 (NCBI Reference Sequence: XM_843653.6):**

1 tgcatggagt ggttgggtgg cagagacgtg ggagtgacca gggttcacgg aggaaggaac

61 caagtgacat cccaagggtt gcataaactc ccaggccgcc aaagaggaca gatactgtgg

121 aacctagaaa ccattgagtg ccgctgtata ggaaagaagc cggagccctt ccccgagccc

181 gctggaacat agaggctcga agcaccgatt gaccatgatg cgcacaccct gcttgctgct

241 gctgctgctg ccgccggtgc tgtgcgtctc tgagacctca ctagagccct gcgaagtaga

301 tgacgacgat ttccgctgct tctgcaactt cacggatccg cagcccgact ggtccagcgc

361 attccagtgt atgattgccg tcgaggtgga gatccacggc ggcggccgca gcctggaaca

421 atttctaaag ggcgcagacg cggacccaaa gcagtacgct gacatggtca gggccctgcg

481 tttgcggcgg ctcaccgtgg cctctgcaca ggttcctgct gtgctggtga ccgccttcct

541 gcgggcgctg gggtactccc gcctcaagga actgacgctg caggacctgg aggtaaccgg

601 cacgccgccg ccgccgcctc tggaagccac tgggcctgcg ctgtccaccc tcaccctccg

661 gaacgtgtcg tgggcaacgg gaagtgcctg gctcgccgaa ctgcagcggt ggctgaagcc

721 gggcctcaag gtactgaaca tcgcgcaggc acactcgctt gctttttcct gcgcacgact

781 ccgcaccttc ccggcgctca ccaccttaga cctatccgac aatcccggac tgggagagca

841 cggactggct tcggctctct gtccgcaaaa gttcccggcc ctccaggctc tcgtcttgcg

901 caacgcggga atgcatacgc cgaacggcgt gtgcgcggcg atggcggtgg cgggtgtgca

961 accccgccac ctagacctca gccacaactc gctgcgcgct accgccccag gcgctcctgc

1021 gtgtgtctgg cccagcgcac tggactctct caacttgtcc ttctctgggc tggggcaggt

1081 gcctaaggga ctacccgcca ggctgagcgt gctcgatctt aggtgcaaca agctgaacag

1141 agagcctcgg ctagaagagc tgcctaaggt gagcaacctg acactggacg ggaatccctt

1201 tctggacccc gaagctccca agtaccaaga aaacccgacg acgtctggcg tggtcccatc

1261 ctgtgcgcgt tcgggcctgg cggtggggat gtcaggaact ctcgcggtgc ttcaaagggt

1321 ggggggcttt gcctgaggcc caggagagac caatgaattg cctcagattg ccctgcttcc

1381 agcaaagccg catcaggacg ttttaaccaa ccaacccgca gccctatctt cattaaaatc

1441 tggaacaacg a
