# Supplementary figures and images for: Clinical anemia predicts dermal parasitism and reservoir infectiousness during progressive visceral leishmaniosis
Source: PLoS Negl Trop Dis. 2024 Nov 8;18(11):e0012363. doi: 10.1371/journal.pntd.0012363 (PMC11578447; doi:10.1371/journal.pntd.0012363)

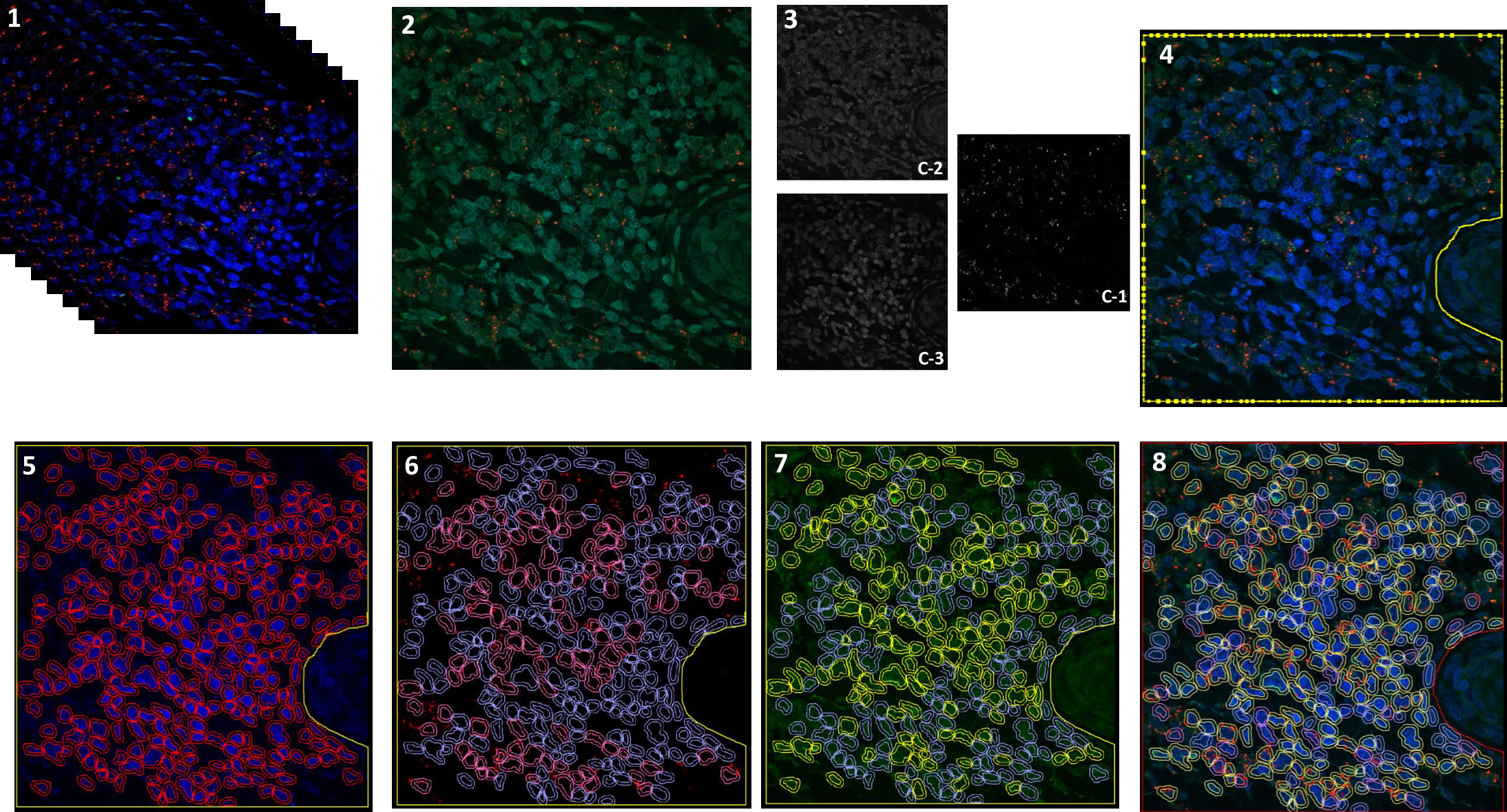

Supplement: S2 Fig — Z-stacks from confocal microscope (1) converted to flax maximum intensity projections in Fiji (2). In Fiji, projection split into C-1, C-2, and C-3 (3). Brightness and contrast were optimized for each channel, and the channels were merged back together. In QuPath, the area of interest in each image was annotated, excluding non-inflammatory structures (4). Cells were counted within the annotation (5), and then separate object classifiers for amastin+ (6) and CD14+ (7) based on maximum signal intensity in a cell. A composite classifier was then created to detect double positive cells (8). Intracellular amastin spots (max size 2μm2) and clusters counted using QuPath’s subcellular spot detection feature to determine the number of dermal parasites (8). (TIF) [file pntd.0012363.s002.tif]
