# Supplementary material for: Clinical anemia predicts dermal parasitism and reservoir infectiousness during progressive visceral leishmaniosis
Source: PLoS Negl Trop Dis. 2024 Nov 8;18(11):e0012363. doi: 10.1371/journal.pntd.0012363 (PMC11578447; doi:10.1371/journal.pntd.0012363)
Supplement: S1 Table — Summary of the age in years, sex, LeishVet status, and 4DX SNAP results for tickborne diseases of all dogs. Multiple* includes 2 animals positive for Borrelia burgdorferi/Ehrlichia and 1 animal positive for Borrelia burgdorferi/Anaplasma. (DOCX) [file pntd.0012363.s003.docx]

|  | LeishVet I  (N = 7) | LeishVet II  (N = 17) | LeishVet III  (N = 6) | LeishVet IV  (N = 7) |
| --- | --- | --- | --- | --- |
| **Age (Years)** |  |  |  |  |
| 1-2 | 3 (42.86%) | 1 (5.88%) | 1 (16.67%) | N/A |
| 3-4 | N/A | 9 (52.94%) | N/A | 2 (28.57) |
| 5-6 | 3 (42.86%) | 5 (29.41%) | 4 (66.67%) | 3 (42.86) |
| 7+ | 1 (14.29%) | 2 (11.76%) | 1 (16.67%) | 2 (28.57) |
| **Sex** |  |  |  |  |
| M | 4 (57.14%) | 13 (76.47%) | 4 (66.67%) | 6 (85.71) |
| F | 3 (42.86%) | 4 (23.53%) | 2 (33.33%) | 1 (14.29) |
| **Tickborne Diseases** |  |  |  |  |
| Negative | 3 | 7 | 3 | 2 |
| *Ehrlichia* | 1 | 9 | 2 | 2 |
| *Borrelia* | 1 | - | - | - |
| *Anaplasma* | - | - | 1 | 1 |
| Multiple* | - | 1 | - | 2 |
