# Supplementary material for: Clinical anemia predicts dermal parasitism and reservoir infectiousness during progressive visceral leishmaniosis
Source: PLoS Negl Trop Dis. 2024 Nov 8;18(11):e0012363. doi: 10.1371/journal.pntd.0012363 (PMC11578447; doi:10.1371/journal.pntd.0012363)
Supplement: S2 Table — N indicates the number of 40X fields analyzed. (DOCX) [file pntd.0012363.s004.docx]

| **Amastin^+^CD14^+^** | | | |
| --- | --- | --- | --- |
|  | LeishVet I  (N = 44) | LeishVet II  (N = 117) | LeishVet III/IV  (N = 81) |
|  |  |  |  |
| **Minimum** | 0 | 0 | 0 |
| **Median** | 3 | 8 | 10 |
| **Maximum** | 18 | 136 | 135 |
|  |  |  |  |
| **Mean** | 4.841 | 18.82 | 16.77 |
| **Std. Deviation** | 4.903 | 26.30 | 21.03 |
| **Amastin^+^CD14^-^** | | | |
|  | LeishVet I  (N = 44) | LeishVet II  (N = 117) | LeishVet III/IV  (N = 81) |
| **Minimum** | 1 | 1 | 0 |
| **Median** | 8 | 14 | 16 |
| **Maximum** | 29 | 92 | 93 |
|  |  |  |  |
| **Mean** | 10.27 | 18.83 | 20.62 |
| **Std. Deviation** | 8.505 | 16.71 | 18.68 |
| **Amastin^-^CD14^+^** | | | |
|  | LeishVet I  (N = 44) | LeishVet II  (N = 117) | LeishVet III/IV  (N = 81) |
|  |  |  |  |
| **Minimum** | 2 | 2 | 5 |
| **Median** | 15.5 | 33 | 31 |
| **Maximum** | 61 | 130 | 125 |
|  |  |  |  |
| **Mean** | 19.91 | 38.73 | 38.90 |
| **Std. Deviation** | 14.72 | 26.88 | 26.28 |
