# Supplementary material for: Clinical anemia predicts dermal parasitism and reservoir infectiousness during progressive visceral leishmaniosis
Source: PLoS Negl Trop Dis. 2024 Nov 8;18(11):e0012363. doi: 10.1371/journal.pntd.0012363 (PMC11578447; doi:10.1371/journal.pntd.0012363)
Supplement: Attachment — Submitted filename: Review for PLOS NTD.docx [file pntd.0012363.s005.docx]

**Review of the Manuscript:**

Based on the article "Clinical anemia predicts dermal parasitism and reservoir infectiousness during progressive visceral leishmaniasis," here are some review comments and suggestions to authors:

Title and Abstract:

The title is clear and accurately reflects the content of the manuscript. It highlights the key finding of the study, which is the relationship between clinical anemia, dermal parasitism, and infectiousness in visceral leishmaniasis (VL). The abstract provides a concise summary of the study. It clearly states the problem, methodology, key findings, and implications. The mention of the significant correlations found between anemia, dermal parasitism, and infectiousness is particularly impactful.

Introduction and Methods:

The introduction effectively sets the stage for the research. It provides sufficient background on VL and the role of dogs as reservoirs. The discussion of the immune response and its relation to clinical signs is well-explained. The rationale for focusing on anemia rather than renal failure is compelling and supported by recent studies.

The methods section is detailed and thorough. The use of RNAscope and confocal microscopy is appropriate for evaluating dermal parasitism. The explanation of the LeishVet clinical stages and the parameters measured (anemia, creatinine) is clear. The inclusion of a CONSORT flow diagram for sample collection is a good practice, enhancing transparency.

Results and Discussion:

The results are well-presented, with clear distinctions made between the different stages of disease and their corresponding levels of dermal parasitism and infectiousness. The significant correlations between anemia and infectiousness are convincingly shown. The lack of correlation with creatinine reinforces the study’s focus on anemia. The discussion aptly interprets the results, emphasizing the importance of anemia in predicting infectiousness. The authors provide a logical argument for why classical measures of disease severity, like renal failure, do not correlate with infectiousness. The implications for public health initiatives are well-articulated, suggesting a shift in focus to early signs of disease.

Conclusion:

The conclusion succinctly summarizes the main findings and their public health implications. The recommendation to focus on anemic dogs for breaking the zoonotic cycle is well-supported by the data.

Minor Points for Improvement:

1. Abstract (Lines 19-41): Consider adding specific data or statistics to support the statements made about correlations between anemia and infectiousness. This will provide a more quantitative summary of the findings

2. Clarity in the Methods Section (Lines 153-166): The manuscript could benefit from a more detailed explanation of the statistical methods used to analyze correlations between infectiousness. This includes specifying the type of correlation analysis (e.g., Pearson, Spearman) and any adjustments made for potential confounders.

3. Discussion and Implications: Public Health Recommendations (this is optional), The manuscript suggests a shift in focus for public health initiatives but could further elaborate on specific strategies that could be implemented based on these findings, such as targeted interventions for dogs with mild disease.

4. Consistency: Ensure consistency in terminology throughout the manuscript (e.g., use of "leishmaniasis" vs. "leishmaniosis").

Overall Impression and Recommendation:

The manuscript provides an insightful exploration into the relationship between clinical anemia and dermal parasitism in dogs infected with *Leishmania infantum*, and its implications for infectiousness to sand flies. The study's focus on anemia as a predictor of infectiousness in dogs with *L. infantum* offers a novel perspective and addresses an important gap in understanding the progression of leishmaniosis that could influence future public health policies targeting zoonotic visceral leishmaniasis (VL). The manuscript is well-written, methodologically sound, and contributes meaningfully to the field, it is suitable for publication in PLOS Neglected Tropical Diseases
