# Supplementary material for: Clinical anemia predicts dermal parasitism and reservoir infectiousness during progressive visceral leishmaniosis
Source: PLoS Negl Trop Dis. 2024 Nov 8;18(11):e0012363. doi: 10.1371/journal.pntd.0012363 (PMC11578447; doi:10.1371/journal.pntd.0012363)
Supplement: Attachment — Submitted filename: PLOS NTD Response to Reviewers.docx [file pntd.0012363.s006.docx]

First, we want to thank all the reviewers for their time and consideration of this manuscript. We appreciate your thoughtful comments and have addressed them below in ways that we feel have strengthened the manuscript to be published in *PLOS Neglected Tropical Diseases*.

# **Reviewer 1**

1. Discussion on the tick coinfection status of these dogs is missing, particularly with respect to the known Ehrlichia endemicity in this cohort and its ability to induce anemia. Whether Ehrlichia serology was performed at the time of skin collection for the cohort should be described and the proportion of positive dogs within this cohort. For the anemia correlations, separating by Ehrlichia+ and Ehrlichia- dogs would be suggested, where Ehrlichia- dog anemia can more strongly be attributed to Leishmania induced bone marrow alterations.

- **Response**: Thank you for the comment on an important component of canine leishmaniosis. Tickborn infections, particularly *Ehrlichia*, do significantly contribute to the progression of canine leishmaniosis, and many dogs in the tested cohort tested positive for *Ehrlichia* or another tickborne infection on 4DX SNAP. We updated the demographics (Table S1) to include the number and percentage of dogs infected with tickborne coinfections for each LeishVet stage. To further investigate the effect of *Ehrlichia* and other tickborne pathogens on the anemia correlations, we ran some additional analysis, which are attached below. While SNAP positive individuals tended to have a more severe anemia, the median counts of infected monocytes were not significantly different between the two groups, based on Mann-Whitney tests. Because *Ehrlichia* and tickborne infection status did not affect the outcomes measured in the paper, we felt that a discussion of these was outside the parameters of this paper.
-

  - Mann-Whitney test: p = 0.8653
-

  - LeishVet 2 only Mann-Whitney test: p = 0.7785
-

  - Tickborne Coinfection (TBC) Mann-Whitney Test: p = 0.6286
-

  - LeishVet 2 TBC Mann-Whitney test: p = 0.3568

2. Figures S3 and S4 are very similar, and I do not see S4 referenced anywhere in the text, so one should be removed. S4 format is preferred showing the individual channels and should be moved to the main text. This figure should also include a representative image set from a LeishVet stage I dog for comparison.

- **Response**: We added in representative images from a LeishVet I dog as a comparison and moved the figure to the main text as Figure 2 (Line 208 – 215). Thank you for the comment that helped better illustrate the microscopy findings from our study.

3. Figure 2 legend, line 197, outliers indicated with X. No X appears in the figure, update.

- **Response**: We have updated the figure legend to correspond to the figure

4. Figure 4-6, the focus on analysis just of infected monocytes leaves out important information you have access to with this data. Clearly amastin is found in CD14- cells, in fact you have higher amastin+CD14- counts than amastin+CD14+ counts in every group, indicating another host cell exists. Therefore total amastin or CD14-amastin+ correlations should also be performed and commented on against all relevant targets. Either add figure panels or state in the text, expand the results to include the correlation results between creatinine and anemia against the other combinations of amastin: total amastin counts (amastin+CD14-/+) or amastin+CD14-.

- **Response**: Thank you for the comment! We are also very interested in the amastin+/CD14- population. The infected CD14^-^ cells could be local dendritic cells or another immune cell subset that does not express CD14. Canine monocytes have also been shown to have pleomorphic expression of CD14. We edited the discussion to address these cells and their possible identity, while also highlighting the need for additional research to better characterize their role in canine leishmaniosis (Line 304 – 311).

5. The discussion of CKD and urea’s potential effects on the immune response and local skin microenvironment are nicely discussed, although an association between CKD and dermal parasite RNA was not found. A significant association was found between anemia and dermal parasite RNA and infectiousness to sand flies, yet the discussion between how anemia could influence the dermal immune environment or sand fly feeding is lacking. Please add some discussion of mechanisms that may link systemic anemia to skin parasitism or increased uptake by sand flies. Relatedly, is anemia correlated with blood or spleen parasite burden in this cohort? That could support the systemic load is higher in dogs with anemia.

- **Response**: The mechanisms that may link systemic anemia to skin parasitism or increased uptake by sand flies is something we want to further characterize in future work. We edited the discussion to more clearly articulate some hypothesized mechanisms, such as increased parasitism of the bone marrow leaded to dispersal of more parasites to the skin (Line 304-305). For the subset of dogs with recorded blood and spleen parasite burden, anemia was not significantly correlated with either blood or spleen parasite burden. However, this could be due to the small sample size.

6. Line 36, the authors statement that anemia influenced infectiousness to sand flies is overinterpreting. A correlation was observed but may be independent. No testing a direct link between anemia and infectiousness to sand flies was performed. Please use correlated to be more appropriate.

- We have replaced “influenced” with “correlated with” as suggested to more accurately characterize the study’s findings.

7. Lines 90-91, the conclusion of that study was not that moderate diseased dogs are more infectious than severely diseased dogs, but that infectiousness to sand flies was most correlated with dermal parasite burden over clinical severity.

- **Response**: Thank you for the comment – we have edited the manuscript to clarify that dermal parasite burden best correlated with infectiousness to sand flies, and that the highest dermal parasite burden was documented in dogs with mild to moderate clinical disease, based on the LeishVet staging system.

8. Figure 2 legend, line 197, outliers indicated with X. No X appears in the figure, update.

- **Response**: We have updated the figure legend to correspond to the figure

9. Canine dermal skin parasitism by Leishmania is thought to be a strong determinant of host infectiousness to sand fly vectors, which spread the parasite to humans. The authors utilize a cutting edge technique, RNAscope, to probe a cohort of retrospective and prospective CanL skin sections to compare dermal Leishmania Amastin RNA and canine CD14 RNA counts between dogs with a range of clinical severity based on the LeishVet staging system. This method performed well on the canine skin samples and demonstrates the strong usefulness of this tool for expanding the ability of the field to explore in situ dermal infection and the skin immune microenvironment in canines. Interesting findings include CD14+ cells are not the only host cell in the skin and that anemia may influence skin parasite load and transmissibility to sand fly vectors. The mechanisms linking anemia to increased skin parasite load, whether a direct mechanism affecting accumulation of parasites in the skin or that anemia is simply a biomarker for increased bone marrow infection and therefore systemic parasite burden, are not fully fleshed out.

- **Response**: Thank you very much for your thoughtful and helpful review of our manuscript. We appreciate your comments and feel that they have helped make our manuscript clearer and more impactful.

# **Reviewer 2**

1. The manuscript provides an insightful exploration into the relationship between clinical anemia and dermal parasitism in dogs infected with Leishmania infantum, and its implications for infectiousness to sand flies. The study's focus on anemia as a predictor of infectiousness in dogs with L. infantum offers a novel perspective and addresses an important gap in understanding the progression of leishmaniosis that could influence future public health policies targeting zoonotic visceral leishmaniasis (VL). The manuscript is well-written, methodologically sound, and contributes meaningfully to the field, it is suitable for publication in PLOS Neglected Tropical Diseases
   - **Response**: Thank you for your kind comments and thoughtful review of our manuscript.

# **Reviewer 3**

1. The methods were appropriate for the study described. In the section starting at line 153 the researchers should restate what the outcome is for the GEE model more specifically than "mean counts." It is not clear if multiple GEE models were run for each of the following outcomes "number of infected monocytes, other infected cells,
   uninfected monocytes, and parasite burden" (stated in lines 150 and 151) or just one model.

- **Response**: We have updated the language to more clearly show that multiple GEE models were run for different outcomes listed (Line 161 – 165). We also expanded on how the GEE model was utilized to analyze the data. Thank you for the comment that allowed us to clarify the statistical methods we used!

2. The researchers should also consider including a supplemental document showing the different LeishVet stages in more detail. The authors reference the clinical stages but a table describing the staging may be more helpful for readers.

- **Response**: Thank you for the comment – we considered adding a supplemental figure describing the different LeishVet stages but decided not to because the stages are already very well-described in LeishVet’s published, open-access guidelines. To help readers that may be less familiar with the LeishVet staging system, we added additional language summarizing the key characteristics of the LeishVet in the introduction (Lines 88-91) where it is first mentioned and cited the LeishVet guidelines for interested readers.

3. At line 22 consider changing "were considered" to "have previously been considered."

- We have updated the wording as suggested

4. At line 31 the authors should change LeishVet 2 to LeishVet II. Roman numerals are used throughout the rest of the manuscript.

- We updated the “2” to Roman numbers to remain consistent with the rest of the manuscript

5. At line 56 please change "influences" to "influence."

- We have updated “influences” to “correlate with” at the suggestion of another reviewer, as it more accurately reflects the study’s findings.

6. Starting at line 89 in the introduction the authors should consider expanding on the LeishVet clinical scoring system. The authors can then elaborate further on the importance of using the LeishVet scores in the methods and highlight how the scoring system provides a more robust understanding of clinical stage and in concordance infectiousness in the discussion section.

- **Response**: Thank you for the comment – we added language in the introduction summarizing the LeishVet staging system (Lines 88-91). Additionally, in the methods section, we added language clarifying why the LeishVet system was used - “to standardize categorization and facilitate discussion of clinical severity” (Line 145-146).

7. At line 122 considering changing text to read "with additional details in Scorza et al."

- We edited the text as suggested to improve clarity.

8. The authors should consider adding additional information in the discussion to highlight the public health impact of developing targeted interventions to reduce disease transmission in dogs with less severe disease. The authors touch on that importance in the introduction but mainly highlight the reasoning behind the higher parasite burden in the discussion. Several sentences on the public health impact of this study would be valuable at the end of the discussion.

- **Response**: Thank you for the comment, which created an opportunity for us to re-address the public health impact of this work and link the discussion to the introduction! At this point, further education of veterinarians and public health officials about the correlation between anemia and infectiousness (particularly in the absence of chronic kidney disease), as well as potential difference between clinical severity and infectiousness would be valuable, given that current public health interventions often focus on clinical severity. We added some language to the discussion articulating this (Line 337 – 344).

**Reviewer 4**
First, I would like to congratulate the authors on the study. It presents methodological rigor, presents defined objectives, has a reasonable study design, complies with ethical requirements and performs correct statistical analysis. Finally, minor modifications should be made to bring more clarity to the study.
1. Onychogryphosis was not mentioned, as this sign/symptom is pathognomonic of canine visceral leishmaniasis. Was this symptom evaluated in the animals observed? If not, justify why it was not observed.

- **Response**: While onychogryphosis is a classic sign of canine leishmaniosis, we do not see it in this population because their housing has concrete floors that wear down the nails. Therefore, most individuals in this population have very short nails, even when they have clinically apparent canine leishmaniosis.

2. What dosage of dexmedetomidine was used? Was the animal's weight assessed? And what was the animal's reaction after anesthesia? I think it is worth detailing the entire anesthetic protocol.

- **Response**: Thank you for the comment – we edited the methods section to include more details about the sedation protocol (Lines 115-120). We followed the manufacturer's published dosing guidelines.

9. The authors used the ELISA and DPP techniques, in addition to RT-qPCR. The ELISA technique is not the gold standard for antigen-antibody evaluation. I recommend including RIFI or justifying why it is not used.

- **Response**: Thank for the comment – because our retrospective samples were tested with DPP and/or ELISA, we used those techniques for all samples to maintain consistency between retrospective and prospective samples. Additionally, we edited the Methods section (Lines 125-126) to clarify that SLA is a quantitative serological test, which is consistent with the most recent LeishVet guidelines for the diagnosis of canine leishmaniosis.

10. The analysis presented corresponds to the analysis plan, with clear and complete results. I recommend evaluating the resolution of Figure 7, as it presents areas of quality blur.

- **Response**: We evaluated the resolution of Figure 7 and uploaded a new file with improved resolution.

11. The study has potential and is a great contribution to the field of leishmaniasis. I recommend that, in addition to the methodology suggestions, the epidemiological aspect be included. For example, the analysis of the clinical characteristics collected was not categorically analyzed. In addition, data collection on associated factors should be included, such as: whether the animal frequently visits the veterinarian, uses any skin care products, the climate/temperature of the place of residence, among others. I am sure that the addition of these analyses will bring more robustness to the study.

- **Response**: Thank you for the comment – the epidemiological aspect is an important aspect of this study. We edited the Methods section (Line 105-106) to describe some of this population's characteristics and reference previous studies, such as Duprey 2006) that described its epidemiology.

## **Reviewer 5 (Word Document)**

1. Abstract (Lines 19-41): Consider adding specific data or statistics to support the statements made about correlations between anemia and infectiousness. This will provide a more quantitative summary of the findings

- **Response**: We added in the Spearman correlation coefficients and p-values for the correlations between anemia and infectiousness, as well as dermal parasitism (Line 33).

2. Clarity in the Methods Section (Lines 153-166): The manuscript could benefit from a more detailed explanation of the statistical methods used to analyze correlations between infectiousness. This includes specifying the type of correlation analysis (e.g., Pearson, Spearman) and any adjustments made for potential confounders.

3. Discussion and Implications: Public Health Recommendations (this is optional), The manuscript suggests a shift in focus for public health initiatives but could further elaborate on specific strategies that could be implemented based on these findings, such as targeted interventions for dogs with mild disease.

- **Response**: Thank you very much for the comment that was also mentioned by another reviewer! Increased education of veterinarians and public health professionals about these correlations between dermal parasitism, infectiousness, and anemia (particularly in the absence of chronic kidney disease), as well as differentiating between clinical severity and infectiousness, would be important to raise awareness. Additionally, further research into the mechanisms connecting anemia, dermal parasitism, and infectiousness is important to identify potentially effective interventions. (Line 337 – 344).

4. Consistency: Ensure consistency in terminology throughout the manuscript (e.g., use of "leishmaniasis" vs. "leishmaniosis").

- **Response**: We double-checked the use of “leishmaniasis” vs “leishmaniosis” throughout the manuscript and added language (Line 43) to clarify that we use “leishmaniasis” for the human disease and “leishmaniosis” for the canine disease.
